# PD-L1 is remarkably over-expressed in EBV-associated pulmonary lymphoepithelioma-like carcinoma and related to poor disease-free survival

**DOI:** 10.18632/oncotarget.5028

**Published:** 2015-08-19

**Authors:** Wenfeng Fang, Shaodong Hong, Nan Chen, Xiaobo He, Jianhua Zhan, Tao Qin, Ting Zhou, Zhihuang Hu, Yuxiang Ma, Yuanyuan Zhao, Ying Tian, Yunpeng Yang, Cong Xue, Yanna Tang, Yan Huang, Hongyun Zhao, Li Zhang

**Affiliations:** ^1^ State Key laboratory of Oncology in South China, Department of Medical Oncology, Sun Yat-Sen University Cancer Center, Guangzhou, P. R. China; ^2^ Collaborative Innovation Center for Cancer Medicine, Sun Yat-sen University Cancer Center, Guangzhou, Guangdong, China; ^3^ Department of Oncology, the Fifth Affiliated Hospital of Sun Yat-sen University, Zhuhai, Guangdong, China

**Keywords:** pulmonary lymphoepithelioma-like carcinoma, PD-L1, Epstein–Barr virus

## Abstract

**Backgroud:**

Programmed cell death-ligand 1 (PD-L1) and driver mutations are commonly seen in non-small-cell lung cancer (NSCLC). However, the prevelance of PD-L1 over-expression and its prognostic value in Epstein–Barr virus (EBV) associated pulmonary lymphoepithelioma-like carcinoma (LELC) remains poorly understood.

**Methods:**

A total of 214 NSCLC patients and 113 surgically treated pulmonary LELC patients were included. Paraffin-embedded tumor sections were stained with PD-L1 antibody. Correlations between PD-L1 expression and clinicopathological features as well as survival outcomes were analyzed.

**Results:**

The frequency of PD-L1 over-expression in NSCLC was 51.4%. No significant association was observed between common driver mutations and PD-L1 over-expression. Remakably, the positive rate of PD-L1 in pulmonary LELC was 74.3%. High PD-L1 expression was associated with impaired diseas-free survival (DFS) compared with low PD-L1 expression (*p* = 0.008). Multivariate analysis shows that PD-L1 expression level, N stage and M stage were independent prognostic factors for DFS. N stage and M stage but not PD-L1 expression level were significantly associated with overall survival (OS).

**Conclusions:**

PD-L1 over-expression was not related to common driver mutations in NSCLC. Pulmonary LELC have remarkably high incidence of PD-L1 expression. PD-L1 was a negative prognostic factor for DFS in surgically resected pulmonary LELC. These findings may provide a rationale for immunotarget therapy in this virus-associated lung cancer.

## INTRODUCTION

Lung cancer is a leading cause of cancer-related mortality worldwide [1]. Non-small-cell lung cancer (NSCLC) accounts for about 85% of all lung cancer cases and most of NSCLC patients are initially diagnosed at an advanced stage [2]. The overall survival (OS) for this population remains very poor. The most acknowledged risk factor for NSCLC is cigarette exposure. However, several driver mutations have recently been reported to cause NSCLC. These genetic abnormalities include epidermal growth factor receptor (EGFR) mutations, anaplastic lymphoma kinase (ALK) rearrangements, and Kirsten rat sarcoma viral oncogene homolog (KRAS) mutations [3, 4]. The recognition of these driver mutations have led to the development and application of specific tyrosine kinase inhibitors (TKIs) that have greatly changed the treatment paradigm of advanced NSCLC.

Another less understood etiology of NSCLC is Epstein–Barr virus (EBV) infection [5]. This rare subtype of NSCLC is called primary pulmonary lymphoepithelioma-like carcinoma (LELC), which is predominantly diagnosed in Southeast Asia [6]. Literature regarding pulmonary LELC is scarce and no large cohort has been reported. Morphologically, pulmonary LELC resembles undifferentiated nasopharyngeal carcinoma (NPC) which is characterized by prominent lymphoid infiltration and positive EBV-encoded RNA (EBER) by in situ hybridization [7]. In spite of the numerous immune cells around tumor foci, cancer cells manage to evade immune elimination and progress. The underlying factors contributing to the anergy of effector immune cells are poorly understood. Due to the very low incidence of pulmonary LELC, the optimal treatment for this virus-associated tumor remains undefined.

Recently, immunotherapy has intensively been studied in a variety of cancers and is emerging as a promising treatment option [8]. Evolutionally, cancer cells could evade host immune system by expressing specific ligands on membrane to down-regulate cytotoxic T lymphocytes through inhibitory pathways which are usually activated upon ligand-receptor interactions. One important immune inhibitory factor is programmed cell death-1 (PD-1), which is expressed on T cells and negatively regulates their activation and proliferation. The major ligand for PD-1, PD-L1, has been reported to be over-expressed on some cancer cells and related to the maintenance of immunosuppressive conditions. *In vitro* studies have shown that driver mutations not only directly promote the proliferation of cancer cells but also indirectly induce immune evasion via the up-regulation of PD-L1 [9]. However, in clinical setting, the association between EGFR mutations and PD-L1 expression in NSCLC is very controversial [10–12]. Also, the relationship between PD-L1 and ALK rearrangements or KRAS mutations is rarely studied. Recently, some studies have also pointed out that virus-associated tumors aberrantly express PD-L1 after interferon gamma is induced during the anti-viral reaction from the host [13–16]. However, little data is available regarding the prevalence and prognostic role of PD-L1 in EBV-related pulmonary LELC.

Therefore, the present study aimed to prospectively explore the association between PD-L1 expression and common driver mutations in NSCLC. Moreover, we investigated the prevalence and prognostic role of PD-L1 in a large cohort of surgically resected pulmonary LELC.

## RESULTS

### Association between PD-L1 expression and clinicopathological parameters, as well as driver mutations in NSCLC

To avoid selection bias, the first cohort prospectively enrolled 214 non-selective NSCLC patients. Baseline characteristics of these patients are presented in Table 1. Median age at diagnosis was 59 years (range, 24–82 years). One hundred and twenty-two (57%) patients were males and 91 (42.3%) patients were smokers. The number of patients diagnosed at stage I, II, IIIA and IIIB-IV were 79 (36.9%), 47 (22.0%), 40 (18.7%) and 48 (22.4%), respectively. The predominant pathological types were adenocarcinoma (162, 75.7%), followed by squamous cell carcinoma (35, 16.4%), pulmonary LELC (11, 5.1%), and large cell carcinoma (6, 2.8%). The cases of EGFR mutations, ALK rearrangements and KRAS mutations were 72 (33.6%), 14 (6.5%) and 21 (9.8%), respectively.

**Table 1:** Baseline characteristics of NSCLC patients in the first cohort and their association with PD-L1 over-expression

Characteristics	Total	PD-L1 negative (%)	PD-L1 positive (%)	*P*-value	Median PD-L1 level (H-score)	*P*-value
**Gender**						
Female	92	54 (58.7)	38 (41.3)	0.010	0	0.019
Male	122	50 (41.0)	72 (59.0)		30	
**Age, years**						
<59	113	58 (51.3)	55 (48.7)	0.398	0	0.411
>=59	101	46 (45.5)	55 (54.5)		20	
**Smoking history**						
Smokers	91	41 (45.1)	50 (54.9)	0.372	30	0.580
Non-smokers	123	63 (51.2)	60 (48.8)		0	
**Family history of cancers**						
Yes	35	16 (45.7)	19 (54.3)	0.709	20	1.000
No	179	88 (49.2)	91 (50.8)		10	
**Pathology**						
Adenocarcinoma	162	84 (51.9)	78 (48.1)	0.034*	0	0.034
SCC	35	17 (48.6)	18 (41.4)		10	
LCC	6	2 (33.3)	4 (66.7)		30	
LELC	11	1 (0.91)	10 (91.9)		150	
**Tumor differentiation**						
Poor	63	18 (28.6)	45 (71.4)	<0.001	60	<0.001
Moderate	127	68 (53.6)	59 (46.4)		0	
Well	24	18 (75.0)	6 (25.0)		0	
**T stage**						
1	47	25 (53.2)	22 (46.8)	0.846	0	0.406
2	120	56 (46.7)	64 (53.3)		20.0	
3	29	15 (51.7)	14 (48.3)		0	
4	18	8 (44.4)	10 (55.6)		20.0	
**N stage**						
0	103	52 (50.5)	51 (49.5)	0.677	0	0.700
1	40	21 (52.5)	19 (47.5)		0	
2	57	26 (45.6)	31 (54.5)		20.0	
3	14	5 (35.7)	9 (64.3)		35.0	
**Stage**						
I	79	40 (50.6)	39 (49.4)	0.548	0	0.730
II	47	25 (53.2)	22 (46.8)		0	
IIIA	40	20 (50.0)	20 (50.0)		10	
IIIB-IV	48	19 (39.6)	29 (60.4)		20	
**Metastasis**						
Yes	37	15 (40.5)	22 (59.5)	0.281	20	0.470
No	177	89 (50.3)	88 (49.7)		0	
**EGFR mutations**						
Exon 19	32	18 (56.2)	14 (43.8)	0.611	0	0.334
Exon 21	40	18 (47.9)	22 (52.1)		20	
No	142	68 (47.9)	74 (42.1)		20	
**KRAS mutations**						
Yes	21	13 (61.9)	8 (38.1)	0.199	0	0.358
No	193	91 (47.2)	102 (52.8)		20	
**ALK rearrangements**						
Yes	14	10 (71.4)	4 (28.6)	0.099*	0	0.167
No	200	94 (47.0)	106 (53.0)		20	
**Known driver mutations**						
Yes	109	60 (57.1)	49 (43.9)	0.055	0	0.101
No	105	44 (40.4)	61 (59.6)		30	

Abbreviations: NSCLC, non-small-cell carcinoma; PD-L1, programmed cell death ligand 1; EGFR, epidermal growth factor receptor; KRAS, Kirsten rat sarcoma viral oncogene homolog; ALK, anaplastic lymphoma kinase; SCC, squamous cell carcinoma; LCC, large cell carcinoma; LELC, lymphoepithelioma-like carcinoma.

Note:

* Fisher's exact test;

For PD-L1 immunohistochemical staining, we defined cases with more than 5% expression as positive ones. Thus, a total of 110 (51.4%) patients were positive for PD-L1 with a median H-score of 15 (range, 0–230). The correlation between PD-L1 expression and patients' characteristics is shown in Table 1. PD-L1 expression was significantly associated with pathological subtype (*p* = 0.034), tumor differentiation (*p* < 0.001) and gender (*p* = 0.010). However, no significant association was observed between PD-L1 expression and age (*p* = 0.398), smoking status (*p* = 0.372), stage (*p* = 0.548), EGFR mutations (*p* = 0.611), ALK rearrangements (*p* = 0.099) or KRAS mutations (*p* = 0.199). The most striking phenomenon was the PD-L1 expression in pulmonary LELC. In the 11 pulmonary LELC patients enrolled, 10 (90.9%) of them demonstrated PD-L1 positivity with a median H-score of 150 (range, 30–230). Pulmonary LELC showed 9 times higher chance of having PD-L1 over-expression than non-LELC did (OR, 10.30; Fisher's exact test, *p* = 0.028). The remarkable phenomenon led us to expand this cohort of patients to study the overall prevalence and prognostic role of PD-L1 in pulmonary LELC.

### PD-L1 expression in pulmonary LELC and its association with patients' characteristics

**Figure 1:**
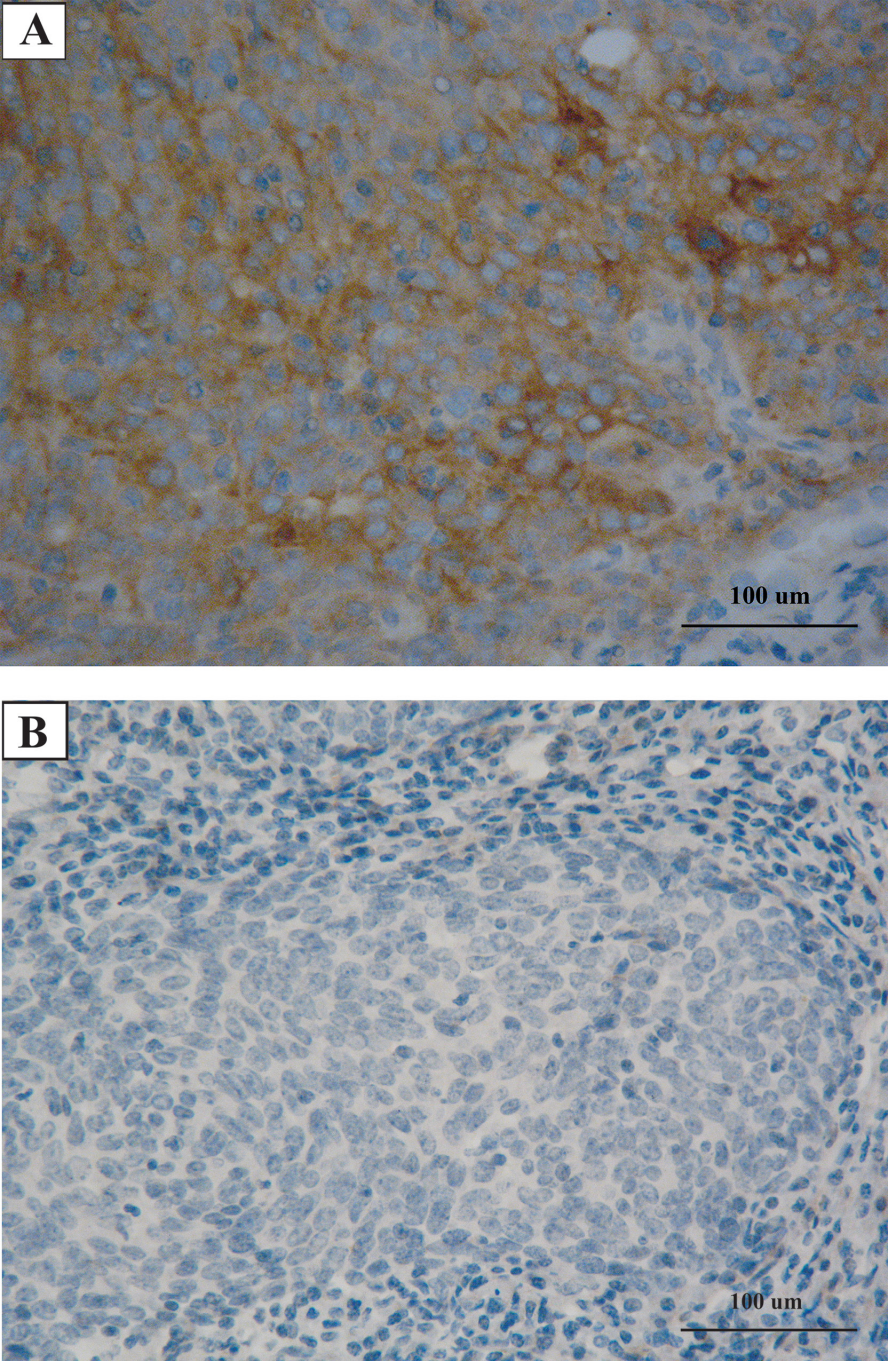
Representatives of programmed cell death-ligand 1 (PD-L1) immunohistochemical staining in pulmonary lymphoepithelioma-like carcinoma **A.** Positive PD-L1 staining with a membranous pattern. **B.** Negative PD-L1 staining in another case. Pulmonary LECL is charaterized by intensive lymphoid infiltration.

The second cohort involved 113 consecutive pulmonary LELC patients who were surgically treated in Sun Yat-sen University Cancer Center. The baseline characteristics of these patients are shown in Table 2. The median age of these patients is 52 years old (range, 28–74 years). Among the 113 patients, 62 (54.9%) were females and 32 (28.3%) were smokers. The patients were pathologically staged as I (29, 25.7%), II (24, 21.2%), IIIA (45, 39.8%) and IIIB-IV (15, 13.3%), respectively. Nine (8.0%) patients received neo-adjuvant chemotherapy and 68 (60.2%) patients received adjuvant chemotherapy. The mutation rate of EGFR gene was 1.8% (2/113). ALK rearrangements and KRAS mutations were not detected. The overall incidence of PD-L1 over-expression was 74.3% (84/113). Representatives PD-L1 staining are shown in Figure 1.

**Table 2:** Baseline characteristics of pulmonary lymphoepithelioma-like carcinoma patients in the second cohort and their association with PD-L1 over-expression

Characteristics	Total	PD-L1 negative (%)	PD-L1 positive (%)	*P* value	Median PD-L1 level (H-score)	*P* value	Low PD-L1 (%)	High PD-L1 (%)	*P* value
**Gender**									
Female	62	17 (27.4)	45 (72.6)	0.638	40.0	0.246	30 (48.4)	32 (51.6)	0.235
Male	51	12 (23.5)	39 (76.5)		60.0		19 (37.3)	32 (62.7)	
**Age, years**									
<52	57	8 (14.0)	49 (86.0)	0.004	50.0	0.041	19 (33.3)	38 (66.7)	0.030
> = 52	5 6	21 (37.5)	35 (62.5)		30.0		30 (53.6)	26 (46.4)	
**Smoking history**									
Smokers	32	10 (31.3)	22 (68.6)	0.393	50.0	0.826	14 (43.8)	18 (56.2)	0.958
Non-smokers	81	19 (23.5)	62 (76.5)		40.0		35 (43.2)	46 (56.8)	
**T stage**									
I	19	5 (26.3)	14 (73.7)	0.027*	50.0	0.085	6 (31.6)	13 (68.4)	0.162*
II	71	17 (23.9)	54 (76.1)		40.0		33 (46.5)	38 (53.5)	
IIIA	13	7 (53.8)	6 (46.2)		0		8 (61.5)	5 (38.5)	
IIIB-IV	10	0 (0)	10 (100)		135.0		2 (43.4)	8 (56.6)	
**N stage**									
0	43	13 (30.2)	30 (69.8)	0.506*	40.0	0.453	20 (46.5)	23 (53.5)	0.842*
1	16	5 (31.2)	11 (68.8)		35.0		8 (50.0)	8 (50.0)	
2	49	11 (23.9)	38 (76.1)		50.0		19 (38.8)	30 (61.2)	
3	5	0 (0)	5 (100)		40.0		49 (43.3)	64 (56.6)	
**M stage**									
0	101	27 (26.7)	74 (73.3)	0.728*	40.0	0.319	45 (44.6)	56 (55.4)	0.337*
1	12	2 (16.7)	10 (83.3)		100.0		4 (33.3)	8 (66.7)	
**Stage**									
I	29	7 (24.1)	22 (75.9)	0.420*	50.0	0.116	12 (41.4)	17 (58.6)	0.290*
II	24	9 (37.5)	15 (62.5)		25.0		14 (58.3)	10 (41.7)	
IIIA	45	11 (24.4)	34 (75.6)		50.0		19 (42.2)	26 (57.8)	
IIIB-IV	15	2 (13.3)	13 (86.7)		130.0		4 (26.7)	11 (73.3)	
**Neo-adjuvant chemo**^$^									
Yes	9	2 (22.2)	7 (77.8)	1.000*	NA	NA	3 (33.3)	6 (66.7)	0.729
No	104	27 (26.0)	77 (74.0)				46 (44.2)	58 (55.8)	
**Adjuvant chemo**									
Yes	68	15 (22.1)	53 (77.9)	0.281	35.0	0.686	33 (48.5)	35 (51.5)	0.173
No	45	14 (31.1)	31 (68.9)		50.0		16 (35.6)	29 (64.4)	
**EGFR mutations**									
Yes	2	0 (0)	2 (100)	1.000*	NA	NA	0 (0)	2 (100)	1.000*
No	111	29 (26.1)	82 (73.9)				29 (26.1)	82 (73.9)	
**KRAS mutations**									
Yes	0	0 (0)	0 (0)	NA	NA	NA	0 (0)	0 (0)	NA
No	113	29 (25.7)	84 (74.3)				29 (25.7)	84 (74.3)	
**ALK rearrangements**									
Yes	0	0 (0)	0 (0)	NA	NA	NA	0 (0)	0 (0)	NA
No	113	29 (25.7)	84 (74.3)				29 (25.7)	84 (74.3)	

Abbreviations: PD-L1, programmed cell death ligand 1; EGFR, epidermal growth factor receptor; KRAS, Kirsten rat sarcoma viral oncogene homolog; ALK, anaplastic lymphoma kinase; SCC, squamous cell carcinoma; LCC, large cell carcinoma; LELC, lymphoepithelioma-like carcinoma; NA, not applicable.

Note:

$ Samples were obtained prior to neo-adjuvant chemotherapy;

* Fisher's exact test; PD-L1 status was determined by the threshold of 5% membrane staining of cancer cells. High and low PD-L1 expression level was defined at the point which best discriminates DFS, namely H-score = 30.

As is shown in Table 2, PD-L1 over-expression was significantly higher in younger patients than that in older patients (86.0% vs 62.5%; OR, 3.68; *p* = 0.004). T stage was also significantly associated with PD-L1 over-expression. Other clinicopathological variables, including gender, smoking history, lymph node stage (N stage), metastasis and pathological stage were not significantly associated with PD-L1 over-expression. Due to the rare mutation rate of EGFR, ALK and KARS, the assessments of the association between PD-L1 over-expression and driver mutations are infeasible.

### Survival analyses of resected pulmonary LELC

**Figure 2:**
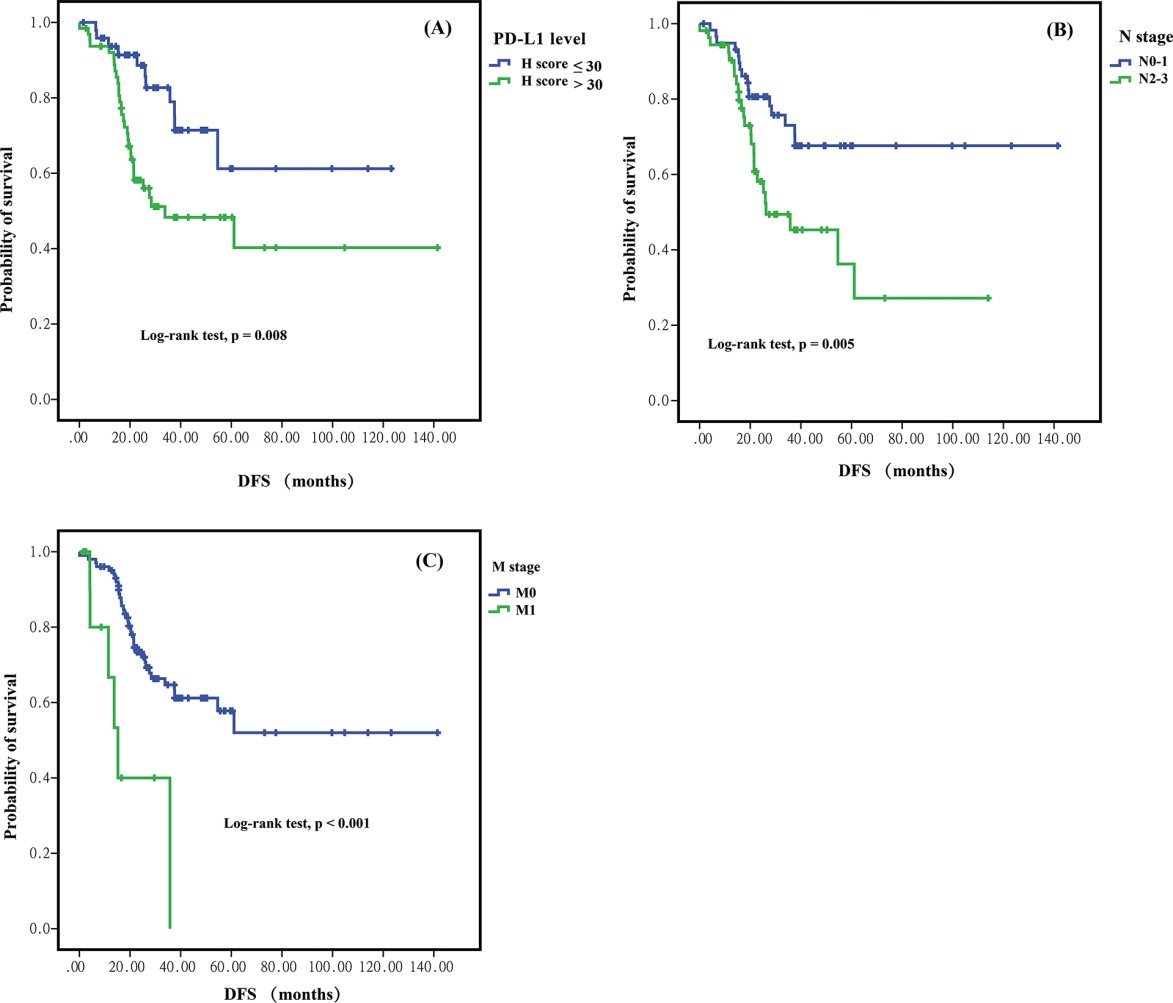
Kaplan-Meier curves for disease-freee survival (DFS) between different groups in sugically resected pulmonary lymphoepithelioma-like carcinom **A.** Higher PD-L1 expression (H-score > = 30), **B.** higher N stage (N2-3), and higher M stage (M1) were significant risk fators for DFS.

**Figure 3:**
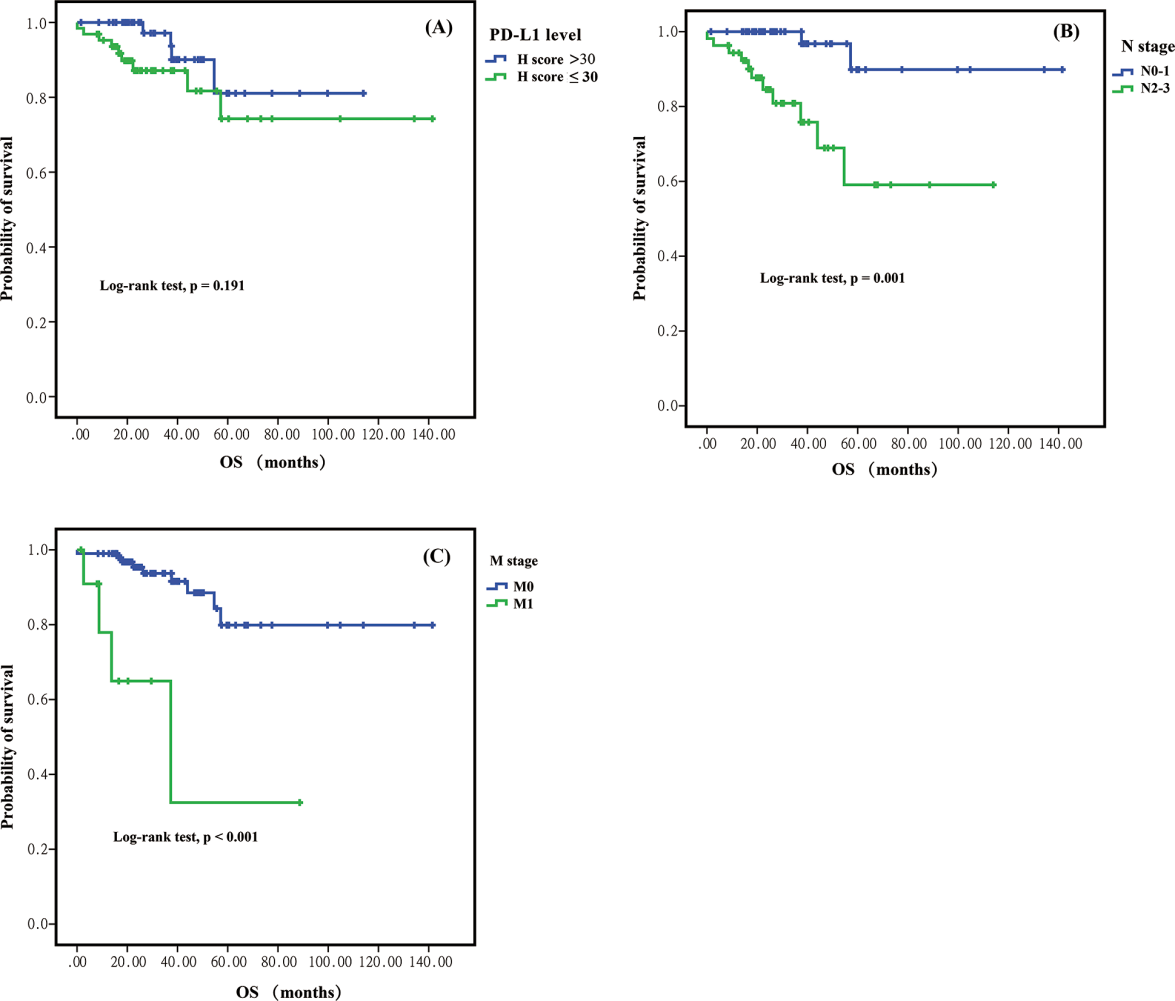
Kaplan-Meier curves for overall survival (OS) between different groups in sugically resected pulmonary lymphoepithelioma-like carcinom **A.** Higher PD-L1 expression (H-score > = 30) was non-significantly associated with poorer OS. **B.** higher N stage (N2-3), and higher M stage (M1) were significant risk fators for OS.

The median follow-up time for DFS and OS was 38.47 and 30.9 months, respectively. A total of 41 DFS events and 13 OS events occurred during the study period. One-, 3- and 5-year DFS were 94.6%, 79.2% and 69.4% respectively. And the 1-, 3- and 5-year OS were 97.3%, 94.3% and 91.4%, respectively. Using X-tile, we determined the best cut-off of PD-L1 H-score for discriminating DFS to be 30, thus dividing patients into those with low PD-L1 expression (H-score < = 30) and those with high PD-L1 expression (H-score > 30). Kaplan–Meier analysis revealed that patients with high PD-L1 expression had shorter DFS than those with low PD-L1 expression (5-year DFS, 48.3% vs 61.2%; *p* = 0.008) (Figure 2). Patients with high PD-L1 expression also tended to have shorter 5-year OS than those with low PD-L1 expression, though the difference was not significant (74.3% vs 81.1%; *p* = 0.191) (Figure 3). In univariate analysis, advanced N stage (N2-3 vs N0-1), advanced M stage (M1 vs M0) and higher PD-L1 expression (H-score, > 30 vs < = 30) was significantly associated with inferior DFS. Only advanced N stage and advanced M stage were significant risk factors for poor prognosis of OS (Table 3). EGFR mutations, KRAS mutations and ALK rearrangements were not analyzed for survival because of the low prevalence.

**Table 3:** Univariate survival analysis of surgically treated pulmonary lymphoepithelioma-like carcinoma patients

Parameters	5-year DFS (%)	*p* value	5-year OS (%)	*p* value
**Age, years**				
<=52	50.5	0.317	78.8	0.783
>52	50.7		75.1	
**Gender**				
Female	57.5	0.153	76	0.936
Male	43.6		77.8	
**Smoking history**				
Smokers	44.2	0.176	74.5	0.304
Non-smokers	59.6		87.5	
**T stage**				
T1-2	58.6	0.136	78.9	0.143
T3-4	28.8		66	
**N stage**				
N1-2	67.6	0.005	89.9	0.001
N3-4	36.2		59.1	
**M stage**				
0	57.8	<0.001	79.9	<0.001
1	NA		32.5	
**Pathological stage**				
I	66.8	0.001	87.5	0.001
II	78.3		92.9	
IIIA	38.4		64.2	
IIIB-IV	NA		36.6	
**PD-L1 status**				
Positive	53.5	0.033	77.6	0.903
Negative	71.8		80.6	
**PD-L1, H-score**				
< = 30	61.2	0.008	81.1	0.191
>30	48.3		74.3	
**Adjuvant chemo**				
Yes	47.4	0.233	76.8	0.562
No	65.9		76.9	
**Neo-adjuvant chemo**				
Yes	NA	0.299	NA	0.751
No	56.1		77.7	

Abbreviations: PD-L1, programmed cell death ligand 1; DFS, disease-free survival; OS, overall survival.

Note: PD-L1 status was determined by the threshold of 5% membrane staining of cancer cells. PD-L1 expression level (defined by H-score) was stratified using X-tile at the cutoff value that best discriminated DFS difference.

In multivariate analysis, high PD-L1 expression, advanced N stage and advanced M stage were independent risk factors for tumor recurrence/metastasis (DFS). For OS, advanced N stage and advanced M stage remained the independent risk factor for poor prognosis (Table 4).

**Table 4:** Cox proportional hazard regression analysis of survival in surgically treated pulmonary lymphoepithelioma-like carcinoma patients

Parameters	Disease free survival		Overall survival	
	HR (95% CI)	*p* value	HR (95% CI)	*p* value
**Age (<=52 vs >52 years)**	0.702 (0.353–1.398)	0.441	0.768 (0.231–2.552)	0.666
**Gender (Female v Male)**	0.657 (0.340–1.271)	0.285	0.905 (0.283–2.897)	0.867
**PD-L1 (High vs Low)**	2.398 (1.196–4.810)	0.014	2.730 (0.756–9.863)	0.125
**N stage (N2-3 vs N0-1)**	2.018 (1.036–3.933)	0.039	6.286 (1.263–31.285)	0.025
**M stage ( M1 vs M0)**	2.944 (1.156–7.497)	0.024	5.052 (1.285–19.863)	0.02

Abbreviations: PD-L1, programmed cell death ligand 1; HR, hazard ratio; CI, confidence interval.

## DISCUSSION

The present study reveals that the overall incidence of PD-L1 over-expression in non-selective NSCLC patients was 51.4% (110/214). There was no significant association between common driver mutations (EGFR mutations, KRAS mutations and ALK rearrangements) and PD-L1 over-expression. However, PD-L1 over-expression was significantly associated with gender, tumor differentiation and pathological types. Remarkably, in pulmonary LELC, a rare subtype of primary lung cancer that is closely related to EBV infection, we found a very high incidence of PD-L1 over-expression (10/11, 90.9%). This phenomenon led us to expand this cohort of lung cancer and to investigate the clinical significance of PD-L1 in pulmonary LELC. In the expanded cohort involving 113 surgical LELC samples, the incidence of PD-L1 over-expression was 74.3%, which was consistent with the study by Chang et al. [17]. High PD-L1 expression (H-score > 30) was associated with impaired DFS compared with low PD-L1 expression (*p* = 0.008). The mutation rate in common driver genes including EGFR, KRAS and ALK was extremely low in pulmonary LELC. These results imply that pulmonary LELC is a very distinct subtype of lung cancer in terms of genetic aberrations and immune status.

The incidence of PD-L1 over-expression in NSCLC has been reported to vary from 19.6% to 65.9% and its association with clinicopathological features as well as patients' prognosis is very controversial [18–21]. In Chen et al.′s study [21], they analyzed 120 NSCLC tissues and found that the incidence of PD-L1 over-expression was 57.5%. No difference in the PD-L1 positive rates was observed among adenocarcinoma, squamous cell carcinoma and large cell carcinoma, whereas, PD-L1 over-expression was significantly associated with poor tumor differentiation, advanced clinical stage and OS. In another study specifically involving 163 stage I lung adenocarcinoma, the incidence of PD-L1 over-expression was 39.9% which was significantly associated with higher tumor differentiation, vascular invasion and better relapse-free survival [12]. In a study including 214 surgically treated squamous cell carcinoma of the lung, the authors concluded that the incidence of PD-L1 over-expression was 19.6% and it was not related to patients' clinical outcomes [18]. Several reasons might contribute to the discrepancy between previous and current studies. First, the baseline characteristics of lung cancer patients among these studies are of great heterogeneity and the difference of PD-L1 expression in different histology and cancer stage may impact the survival analysis. Second, the technique and protocol for PD-L1 detection may differ among these studies. The impact of inter-laboratory variability on the intrinsic association between PD-L1 expression and clinicopathological features, as well as survival outcomes may be difficult to evaluate and clarify. Third, the optimal thresholds for PD-L1 positivity in terms of prognosis discrimination remains undefined and also differ among these studies. In the present study, we defined positive PD-L1 expression as more than 5% of tumor cell staining, the same criteria as that in clinical trials [22]. Also, we used a semi-quantifying tool, H-score which combines percentage and intensity of tumor cell staining to better reflect the expression level of PD-L1 protein. In this way, we were able to dig out the best prognostic value of PD-L1 in pulmonary LELC.

PD-1/PD-L1 pathway has been recognized as a key mediator of immune suppression [23]. Cancer cells could eventually evade host immune clearance by expressing certain ligands to down-regulate cytotoxic T lymphocytes through inhibitory pathways, which are usually mediated by ligand-receptor interactions. PD-1 is mainly expressed on the surface of effector T lymphocytes, while its major ligand, PD-L1 is expressed in a variety of immune cells and cancer cells [24]. PD-1/PD-L1 are recognized as important inhibitory checkpoints for T-cell activation at its maintenance stage [25]. Specific inhibitors targeting PD-1/PD-L1 axis have been promising in breaking off the inhibitory status of host immune system and boosting its anti-cancer power in numerous cancer types [26]. Human anti-PD-1 or anti-PD-L1 antibodies result in durable tumor response and significantly improve survival in advanced melanoma [27], renal cell carcinoma [28] and NSCLC [29]. However, the molecular mechanism of PD-L1 over-expression in cancer cells remains not fully understood. Pardoll et al. proposed that two major mechanisms of PD-L1 regulation may be accountable [8]. The first one is called innate immune resistance by which the constitutive oncogenic activation of signaling pathways not only directly enhance the proliferation of cancer cells but also transcriptionally up-regulate PD-L1 expression to promote immune suppression. Our previous studies have revealed that EGFR mutations could up-regulate the expression of PD-L1 via p-ERK1/2/p-c-Jun pathways [9], while LMP-1, an important oncogene in nasopharyngeal carcinoma could also induce the expression of PD-L1 through STAT3, AP-1, and NF-κB pathways [16]. These *in vitro* studies provide solid evidence that PD-L1 could be regulated by oncogenic pathways. However, in tissue level, the association between PD-L1 and driver mutations is rather controversial. In the present study, we found no significant association between PD-L1 expression and EGFR mutations, KRAS mutations or ALK rearrangements, consistent with a previous study by Yang et al [12]. However, D'Incecco and colleagues found that PD-L1 over-expression was significantly higher in patients with KRAS mutations or EGFR mutations than those with wild type driver genes [11]. One possible explanation is that tumor tissues have far more complicated genetic background and immune contents in the tumor microenvironment than in co-culture experiments. The interactive networks between cancer cells and stroma cells call for *in vivo* studies on the association between oncogenes and PD-L1 regulation. In addition, the expression patterns of PD-L1 deserve deeper investigation. Yet some studies have shown that PD-L1 was positively associated with driver mutations in NSCLC samples [11, 30]. The difference between the present and previous studies may be due to some reasons. First, the sample size varied across different studies and all data were retrospectively collected, leading to potential bias. Second, the threshold of positive PD-L1 expression was also different from each other in these studies. Third, the specificity and reproducibility of the commercially available antibodies has not been thoroughly assessed.

Another mechanism of PD-L1 up-regulation is called adaptive immune resistance. In cancers with chronic virus infection, the anti-cancer and/or antiviral immune response could induce the secretion of important inflammatory factors like interferon gamma (IFN-γ), which may be utilized by cancer cell itself to maintain immune suppressive milieu. Our previous study have indicated that the up-regulation of PD-L1 in EBV infected NPC was partly mediated by IFN-γ. Pulmonary LELC is a rare subtype of primary lung cancer that is morphologically indistinguishable from NPC. It is also closely related to EBV infection indicated by positive in situ hybridization of EBERs and is characterized by intensive lymphoid infiltration. Unlike lung adenocarcinoma, pulmonary LELC rarely presents with EGFR mutations, KRAS mutations or ALK rearrangements. In a study by Liang et al. [31], they found that none of 11 pulmonary LELC harbored EGFR mutations while in a more recent study by Chang et al. [17], the mutation rate of EGFR gene was reported to be 12.5% (8/64). In the present study, we found a remarkably high incidence of PD-L1 expression in pulmonary LELC, further confirming that virus-associated cancers have predominant PD-L1 expression. Indeed, our previous study found that PD-L1 positive rate in EBV-infected NPC samples was as high as 95%. Higher PD-L1 expression independently predicted poorer DFS in NPC patients after definitive radiotherapy. In the present study, we also found that higher PD-L1 expression was significantly associated with shorter DFS independent of N stage, M stage and other clinical variables in surgically treated pulmonary LELC patients. One possible explanation is that residual cancer cells with higher PD-L1 expression are more resistant to immune elimination and have bigger chancer of early recurrence and/or metastasis. Unfortunately, the data for OS was immature in the present study, making it infeasible to assess the prognosis value of PD-L1 in pulmonary LELC.

All in all, we show here that PD-L1 expression was not related to common driver mutations in NSCLC tissues and PD-L1 over-expression is a hallmark of pulmonary LELC. We further propose that the PD-1/PD-L1 axis might play a critical role both in the persistent infection of EBV and the resistance to immune elimination during malignant progression in pulmonary LELC. These findings extend those recently reported in NPC, hepatocellular carcinoma, which are virus-associated cancers and has also been considered as “immunogenic.” Our studies support a rationale for administering anti-PD-1 and anti-PD-L1 therapies to the EBV infected pulmonary LELC population. Future studies will need to characterize the genomic signatures of this tumor and identify additional factors responsible for inducing PD-L1 expression and hence immunosuppression within the tumor microenvironment.

## PATIENTS AND METHODS

### Patients

This study enrolled two cohorts of NSCLC patients. The first cohort prospectively enrolled newly diagnosed NSCLC patients in Sun Yat-sen University Cancer Center (SYSUCC) from January 2014 to December 2014. Patients who were pathologically diagnosed as NSCLC and provided sufficient tumor tissue for genomic analysis were included. The second cohort focused on surgically resected pulmonary LELC. NSCLC patients who were surgically treated in SYSUCC between January 2008 and December 2012 were retrospectively reviewed. Those who were diagnosed as pulmonary LELC with positive EBER results were eligible. All patients underwent endoscopic examination of the nasopharynx to rule out metastatic NPC. Detailed process of patient selection is shown in Figure S1. The study was approved by the Institutional Review Board of SYSUCC and written informed consent was obtained before samples were collected.

### Clinicopathological data

Patients' medical records were reviewed for the collection of clinicopathological information including gender, age, smoking status, tumor size, staging and treatments. Pathologic or clinical staging was determined according to the seventh edition of American Joint Committee on Cancer (AJCC)/International Union against Cancer (IUCC) staging system, which is based on tumor size, location, and the extent of lymph node or distant metastases. OS was defined as the time between surgery and death from any causes. Disease-free survival (DFS) was defined as the time from surgery until recurrence or death. Patients were censored at their last known alive date. Follow-up information was obtained from patients' medical records or by telephone interview.

### Immunohistochemistry analyses

Immunohistochemical staining was performed using rabbit monoclonal anti-PD-L1 antibody (E1L3N™, Cell Signaling Technology, Danvers, MA; dilution 1:200) to determine the expression of PD-L1. Four-μm-thick sections from formalin-fixed, paraffin-embedded (FFPE) tissue blocks were de-waxed and rehydrated accordingly. For antigen retrieval, slides were heat-treated for 30 minutes in citrate buffer solution (p*H* = 7.4) and cooled slowly at room temperature for 20 minutes. After blocking the activity of endogenous peroxidase with 3% hydrogen peroxide for 8 minutes, the sections were treated with anti-PD-L1 antibody and incubated overnight. Subsequently, the slides were rinsed in PBS three times and incubated in HRR-linked secondary antibodies. After incubation, slides were washed again with PBS and then visualized using diaminobenzidine. The sections were counterstained with hematoxylin and then mounted.

Two pathologists blinded to patients' information independently assessed the expression of PD-L. Semi-quantitative H score were determined by multiplying the percentage of positively stained cells by an intensity score (0, absent; 1, weak; 2, moderate; and 3, strong). Cases with more than 5% expression of PD-L1 were considered positive according to previous reports.

### Genetic analysis

EGFR mutations and KRAS mutations were detected using PCR-based direct sequencing. Briefly, genomic DNA was extracted from tumors embedded in paraffin blocks. PCR amplification was done using HotStarTaq DNA polymerase (Qiagen Inc., Valencia, CA) using specific primers. PCR products were sequenced directly using Applied Biosystems PRISM dye terminator cycle sequencing method (Perkin-Elmer Corp., Foster City, CA) with ABI PRISM 3100 Genetic Analyzer (Applied Biosystems, Foster City, CA). ALK rearrangements were detected by Fluorescence in situ Hybridization (FISH) using a break-apart probe to the ALK gene (Vysis LSI ALK Dual Color, Break Apart Rearrangement Probe; Abbott Molecular) per manufacturer's instructions. At least 100 representative tumor cells were counted and then analyzed by using an Olympus fluorescence microscope equipped with orange, green, and 4′, 6-diamidino-2-phenylindole filters. Images were captured using the Video Test Image Analysis System. FISH-positive cases were defined as ≥ 15% of the tumor cells that showed a split red and green signal and/or an isolated (single) red signal. Otherwise, the specimen was classified as ALK FISH negative.

### Statistical analyses

All the statistical analyses were performed using SPSS version 22.0 (SPSS, Inc., Chicago, IL.). Pearson's chi-squared test or Fisher's exact test was used to assess the correlations between PD-L1 expression and categorical variables. Median H-scores of PD-L1 was compared between groups using Mann-Whitney U test. The survival analysis was performed with Kaplan–Meier method and log-rank test was used to evaluate survival difference. Subsequently, the PD-L1 expression status was entered into multi-variate Cox regression analysis. Hazard ratios (HR) and 95% confidence intervals (CI) were used to determine the prognostic value of PD-L1 expression. A two sided *p*-value < 0.05 was deemed significant. Optimal cutoff value of PD-L1 H-score for survival discrimination was determined by X-tile software (Yale University, New Haven, CT, USA) as previously described [32].

The sample size of the first cohort was calculated prior to the initiation of the present study. Sample size was calculated to obtain an accurate estimate of the proportion of patients with PD-L1 positive tumors. Assuming a percentage of 50%, more than 193 samples were required to ensure a 95% CI of less than ± 7% (Wilson score method). Taking into account patients with tumors of undetermined status, an overall sample size of 214 was chosen.

## SUPPLEMENTARY FIGURE


