# Supplementary material for: PD-L1 is remarkably over-expressed in EBV-associated pulmonary lymphoepithelioma-like carcinoma and related to poor disease-free survival
Source: Oncotarget. 2015 Aug 19;6(32):33019–32. doi: 10.18632/oncotarget.5028 (PMC4741746; doi:10.18632/oncotarget.5028)
Supplement: Supplementary file 1 [file oncotarget-06-33019-s001.pdf]

SUPPLEMENTARY FIGURE

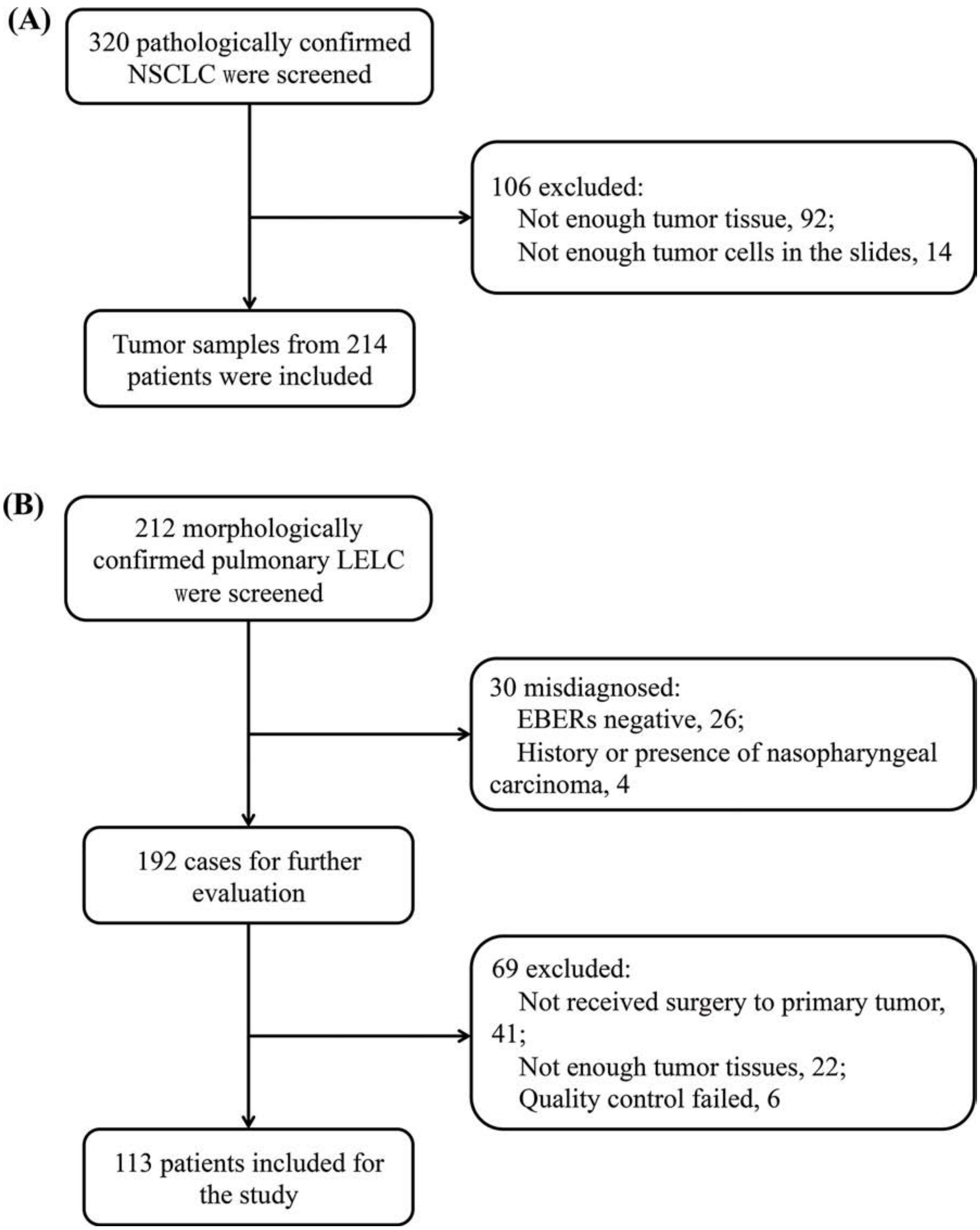

Supplementary Figure S1: Patients selection process of A. the first cohort and B. the second cohort.
